# Metal‐Free Perovskite Piezoelectric Nanogenerators for Human–Machine Interfaces and Self‐Powered Electrical Stimulation Applications

**DOI:** 10.1002/advs.202105974

**Published:** 2022-04-20

**Authors:** Han‐Song Wu, Shih‐Min Wei, Shuo‐Wen Chen, Han‐Chi Pan, Wei‐Pang Pan, Shih‐Min Huang, Meng‐Lin Tsai, Po‐Kang Yang

**Affiliations:** ^1^ Department of Materials Science and Engineering National Taiwan University of Science and Technology Taipei City 10607 Taiwan; ^2^ Department of Biomedical Sciences and Engineering National Central University Taoyuan City 32001 Taiwan; ^3^ National Laboratory Animal Center National Applied Research Laboratories Taipei City 11571 Taiwan

**Keywords:** metal‐free perovskites, piezoelectric nanogenerators, human–machine interfaces, cytotoxicity, electrical stimulation

## Abstract

Single crystal metal‐free halide perovskites have received great attention in recent years owing to their excellent piezoelectric and ferroelectric properties. However, the nanotoxicity and piezoelectricity within the nanoscale of such materials have yet been reported for the demonstration of practical applications. In this work, the observation of intrinsic piezoelectricity in metal‐free perovskite (MDABCO‐NH_4_I_3_) films using piezoresponse force microscopy (PFM) is reported. A cytotoxicity test is also performed on MDABCO‐NH_4_I_3_ to evaluate its low‐toxic nature. The as‐synthesized MDABCO‐NH_4_I_3_ is further integrated into a piezoelectric nanogenerator (PENG). The MDABCO‐NH_4_I_3_‐based PENG (MN‐PENG) exhibits optimal output voltage and current of 15.9 V and 54.5 nA, respectively. In addition, the MN‐PENG can serve as a self‐powered strain sensor for human–machine interface applications or be adopted in in vitro electrical stimulation devices. This work demonstrates a path of perovskite‐based PENG with high performance, low toxicity, and multifunctionality for future advanced wearable sensors and portable therapeutic systems.

## Graphical abstract

Here, the integration of metal‐free perovskite MDABCO‐NH_4_I_3_ into a piezoelectric nanogenerator (MN‐PENG) device is demonstrated. The high‐performance, low‐toxic, and flexible MN‐PENG device shows great potential in biomechanical energy harvesting, human–machine interfaces, and electrical stimulation therapy applications.

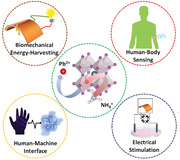

## 1 Introduction

Halide perovskites have evoked significant interests due to their impressive photovoltaic properties, sensing capability, and unique optical versatility.^[^ ^1^, ^2^, ^3^, ^4^ ^]^ The tunable structural diversity has also enabled halide perovskites to exhibit a wide range of promising applications including light emitting diodes, memory devices, nonlinear‐optical devices, and spintronic devices.^[^ ^5^, ^6^, ^7^, ^8^ ^]^ In addition, halide perovskites have been recently proposed to possess excellent piezoelectricity by adopting noncentrosymmetric structures, which is beneficial for designing next generation piezoelectric devices such as piezoelectric nanogenerators (PENG) and piezoelectric sensors.^[^ ^9^, ^10^, ^11^ ^]^ The first halide perovskite PENG was demonstrated in a MAPbI_3_ (MA^+^ = methylammonium) based PENG that exhibit output voltage and current density up to 2.7 V and 240 nA cm^–2^, respectively, after effective polarization.^[^ ^9^ ^]^ However, one of the major challenges for halide perovskite‐based PENG is their relatively low output performance for practical applications.^[^ ^12^ ^]^ Therefore, various strategies including field induced lattice extension, metal substitution, halide incorporation, and organic molecule modification have been applied to improve the output performance of halide perovskite‐based PENG.^[^ ^11^, ^13^, ^14^, ^15^, ^16^, ^17^, ^18^, ^19^ ^]^ However, the toxicity issue in most of the lead halide perovskite PENGs remains a challenge that should be overcome in biomedical related applications, such as cardiac pacemakers, in‐vivo blood pressure monitoring device, wound healing, and nerve restoration.^[^ ^20^, ^21^, ^22^, ^23^, ^24^ ^]^

Previously, several attempts have been made to replace lead ion with tin (II) to achieve an eco‐friendly halide perovskite PENG.^[^ ^10^, ^25^, ^26^ ^]^ However, device instability still remains a critical issue to be solved.^[^ ^27^, ^28^, ^29^ ^]^ Fortunately, metal‐free halide perovskites have been recently predicted to exhibit excellent piezoelectricity, good stability, and low toxicity. In recently reported metal‐free halide pervoskites, highly tunable organic ions and ammonium are utilized to replace metal ions in the perovskite structure.^[^ ^30^, ^31^ ^]^ For example, single crystalline *N*‐methyl‐*N*′‐diazabicyclo [2.2.2] octonium–ammonium triiodide (MDABCO‐NH_4_I_3_) has been reported to possess excellent spontaneous polarization and piezoelectricity due to dipole moments induced by molecular modification.^[^ ^31^, ^32^ ^]^ However, the demonstrations of such materials for various wearable electronics and biomedical applications are still limited.

Herein, we report the first metal‐free MDABCO‐NH_4_I_3_ perovskite PENG (MN‐PENG) for self‐powered sensing, human–machine interface, and electrical stimulation applications. The nanotoxicity of MDABCO‐NH_4_I_3_ has been evaluated by using cell culture with L929 fibroblasts. The piezoelectric properties of MDABCO‐NH_4_I_3_ have been characterized by piezoelectric force microscopy (PFM). Moreover, the as‐fabricated MN‐PENGs have been integrated to realize a smart gesture recognition system. Finally, the MN‐PENGs have been applied to cell proliferation and migration tests to further estimate their feasibility as an in vitro electrical stimulation device. The metal‐free, stable, toxic‐free, and flexible MN‐PENG reported herein provides the potential for the future development of wearable, smart, and portable therapeutic devices.

## 2 Results and Discussion

**Figure 1:**
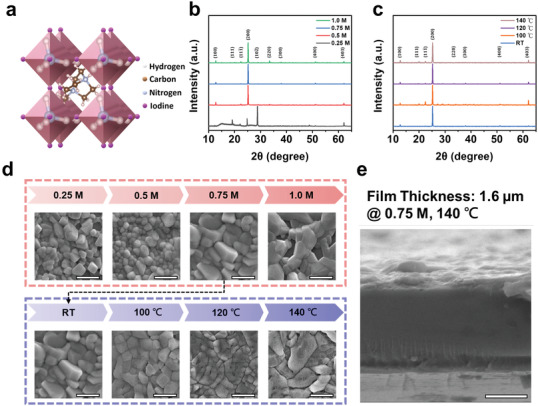
Characterization of MDABCO‐NH_4_I_3_ films. a) Schematic illustration of the MDABCO‐NH_4_I_3_ structure. b) XRD patterns of the MDABCO‐NH_4_I_3_ films with various precursor concentrations. c) XRD patterns of the MDABCO‐NH_4_I_3_ films with various preheating temperature. The precursor concentration is fixed at 0.75 m. d) SEM top view images of MDABCO‐NH_4_I_3_ films prepared with various precursor concentrations and temperatures. e) Cross‐sectional SEM image of the MDABCO‐NH_4_I_3_ film prepared with precursor concentration of 0.75 m and preheating temperature of 140 °C (scale bar = 1 µm).

The schematic illustration of metal‐free perovskite MDABCO‐NH_4_I_3_ structure is shown in **Figure** **1**a. This ABX_3_ structure is composed of A‐site MDABCO^2+^ cation, B‐site NH_4_^+^ cation, and X‐site halide ions. The as‐synthesized MDABCO‐NH_4_I_3_ film was coated on the polyimide substrate with poly(3,4‐ethylenedioxythiophene) polystyrene sulfonate (PEDOT:PSS) as the conductive layer. Detailed experimental procedures are included in the Experimental Section. To optimize the quality of the MDABCO‐NH_4_I_3_ film, we have performed X‐ray diffraction (XRD) and scanning electron microscopic (SEM) characterizations for MDABCO‐NH_4_I_3_ films with different precursor concentrations and preheating temperatures. Figure 1b shows the XRD patterns of MDABCO‐NH_4_I_3_ films prepared with different precursor concentrations (1.0, 0.75, 0.5, and 0.25 m). The characteristic peaks at 12.3°, 19.5°, 21.9°, 24.8°, 28.6°, 33.7°, 37.5°, 50.6°, and 61.9° correspond to the crystal planes of (100), (111), ($11\bar{1}$), (200), ($10\bar{2}$), (220), (300), (400), and (403), respectively. The peak intensity of (200) increases with the precursor concentration, which can be attributed to the preferred orientation induced by high precursor concentration during the growth process.^[^ ^33^, ^34^ ^]^ Figure 1c shows the XRD patterns of the thin film prepared by different preheating temperatures (room temperature, 100, 120, and 140 ℃).^[^ ^31^, ^33^ ^]^ The full width at half maximum (FWHM) of the peak (200) decreases with increased preheating temperature, indicating the formation of grains with larger grain size (Table S1, Supporting Information).^[^ ^35^ ^]^ Additionally, differential scanning calorimetry (DSC) measurement is conducted to understand the structural phase transition of MDABCO‐NH_4_I_3_ films, as shown in Figure S1 (Supporting Information). In Figure 1d, the scanning electron microscopy (SEM) images of MDABCO‐NH_4_I_3_ films prepared by different precursor concentrations and preheat temperatures are presented. It can be observed that the grain size of MDABCO‐NH_4_I_3_ film is significantly enhanced with increased precursor concentration. The results can be attributed to the positive dependence of grain size on the level of supersaturation in one‐step growth mechanism.^[^ ^36^ ^]^ However, it is shown that the surface of MDABCO‐NH_4_I_3_ film becomes relatively nonuniform at the precursor concentration of 1.0 m. The reduced uniformity can possibly attributed to the viscosity increase with increased precursor concentration.^[^ ^37^ ^]^ Therefore, the MDABCO‐NH_4_I_3_ film prepared with 0.75 m precursor concentration was selected for device fabrication by considering the trade‐off between supersaturation and viscosity.^[^ ^34^, ^38^, ^39^ ^]^ In addition, it is also observed that MDABCO‐NH_4_I_3_ film with preheating temperature of 140 ℃ shows the optimal distribution of compact and large grains. The results correspond to the previous reported Volmer–Weber growth process that small grains are grown at low temperature, while large grains are formed at higher temperature.^[^ ^40^, ^41^ ^]^ The cross‐sectional SEM image of MDABCO‐NH_4_I_3_ film with precursor concentration of 0.75 m and preheating temperature of 140 ℃ is shown in Figure 1e. The MDABCO‐NH_4_I_3_ film exhibits a uniform thickness of ≈1.6 µm without any obvious pinholes.

**Figure 2:**
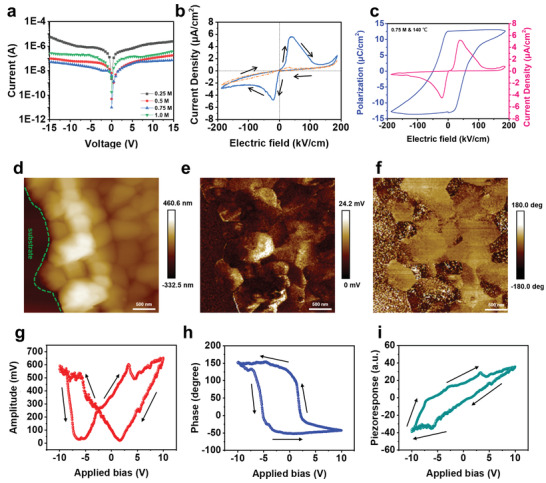
Piezoelectric properties of the MDABCO‐NH_4_I_3_ film. a) Leakage currents with various precursor concentrations. b) Primary (blue) and secondary (red) current density‐electric field measurements by the double‐wave method (voltage sweep rate = 3.33 V s^–1^). c) *P*–*E* curve measured by the double‐wave method. d) Topography image. e) Amplitude image. f) Phase image. g) Amplitude response loop. h) Phase response loop. i) Piezoresponse loop.

To characterize the dependence of precursor concentration on MDABCO‐NH_4_I_3_ film quality, the leakage characteristics of the MDABCO‐NH_4_I_3_ thin film with various precursor concentrations and a preheating temperature of 140 ℃ have been measured and shown in **Figure** **2**a. The sample with precursor concentration of 0.75 m exhibits the lowest leakage current. The leakage current formation has been reported to be strongly related to pinholes, grain boundaries, dangling bonds, and vacancies.^[^ ^42^, ^43^ ^]^ The sample with a precursor concentration of 0.75 m has relatively large grain size and less pinholes (Figure 1d), leading to lower leakage current. In Figure 2b, the polarization‐electric field curve (P‐E curve) of the MDABCO‐NH_4_I_3_ thin film with precursor concentration of 0.75 m and preheating temperature of 140 ℃ has been obtained by the double‐wave method.^[^ ^44^, ^45^ ^]^ The blue curve corresponds to the dipole switching behavior in the first sweep, while the red curve corresponds to the nonferroelectric response in the second sweep. The ferroelectric current–voltage (*I*–*V*) and *P*–*E* curves in Figure 2c can be calculated from Figure 2b, where the values of polarization and coercive electric fields are estimated to be 13.3 µC cm^−2^ and 30 kV cm^−1^, respectively. PFM analysis has been carried out to further confirm the origin of piezoelectricity in the MDABCO‐NH_4_I_3_ film. Figure 2d–f shows topography, amplitude, and phase images from the PFM analysis. The amplitude image clearly shows the piezoelectric response, while the phase image indicates the significant distribution of ferroelectric domains correlated to the grains of the as‐synthesized MDABCO‐NH_4_I_3_ film. The grain sizes of the MDABCO‐NH_4_I_3_ film range from 500 nm to few micrometers.

To further characterize the piezoresponse of the MDABCO‐NH_4_I_3_ film, the amplitude and phase response loops have been obtained by applying dc bias from −10 to +10 V and shown in Figure 2g,h, respectively. The butterfly shaped amplitude loop (Figure 2g) indicates the electrostriction induced by the inverse piezoelectric effect. The two transition points near the bottom of the loop represent the dipole switching behavior. The slight offset near the center of the loop reveals that there exists a built‐in field within the film generated by the spontaneous polarization.^[^ ^26^, ^46^, ^47^ ^]^ The phase response loop (Figure 2h) shows a phase switching behavior of about 180°. The results clearly indicate the polarization change under electric field and the existence of intrinsic ferroelectricity in the MDABCO‐NH_4_I_3_ film. Phase distribution and corresponded phase profile between different ferroelectric domains was also shown in Figures S2 and S3 (Supporting Information), respectively. In addition, the piezoresponse hysteresis loop (Figure 2i) represents the piezoelectric response varied with dipole direction, which can be calculated via Equation (1) below^[^ ^26^, ^48^ ^]^

(1) $$P\left(E\right)=A\left(E\right)\cos\left[\varphi \left(E\right)\right]$$

In Equation (1), *P*(*E*) is the piezoresponse, *A*(*E*) is the amplitude, and *φ*(*E*) is the phase degree, respectively. The piezoelectric coefficient (*d*_33_) can also be estimated by using static‐sensitivity‐based quantification method.^[^ ^49^, ^50^ ^]^ The details of calculation are shown in Equation S1 (Supporting Information). The average value of *d*_33_ is around 12.81 pm V^−1^, similar to the value of MDABCO‐NH_4_I_3_ single crystal reported previously (Table S2, Supporting Information).^[^ ^31^ ^]^ Since the dielectric constant is another key factor for evaluating piezoelectric properties, the relative dielectric constant (*ε*_r_) and the dissipation factor of the as‐synthesized MDABCO‐NH_4_I_3_ film have also been measured (Figure S4, Supporting Information).^[^ ^51^ ^]^

**Figure 3:**
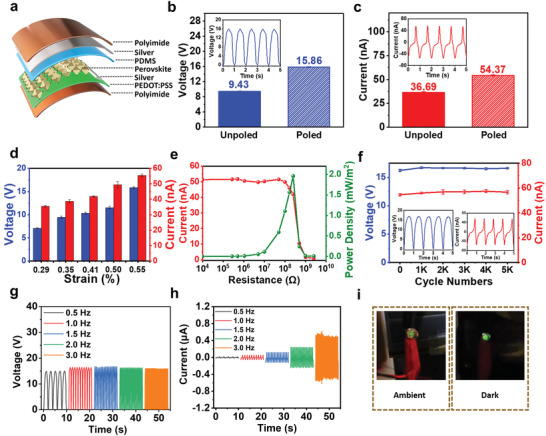
Fabrication and characterizations of the MN‐PENG device. a) Schematic illustration of the configuration of MN‐PENG. b) *V*_OC_ of poled and unpoled devices and voltage waveforms generated by the MN‐PENG (inset). c) *I*_SC_ of poled and unpoled devices and current waveforms generated by the MN‐PENG (inset). d) Output performances of the MN‐PENG plotted as a function of applied strain. e) Output current and power under various external load resistances. f) Output voltage and current under 5000 cycles and waveforms at the end of 5000 cycles (inset). g) Output voltage under different frequencies. h) Output current under different frequencies. i) LED light up by bending the MN‐PENG under 3 Hz.

To realize the MDABCO‐NH_4_I_3_ film in practical applications, we have designed a MN‐PENG using the MDABCO‐NH_4_I_3_ film. A schematic illustration of the MN‐PENG is shown in **Figure** **3**a, where the vertical device configuration is implemented with polydimethylsiloxane (PDMS) as a surface adhesion and passivation layer.^[^ ^9^, ^11^ ^]^ The detailed fabrication procedure of the MN‐PENG is illustrated in Figure S5 (Supporting Information) and the Experimental Section. To obtain the optimal device performance for the MN‐PENG, the MDABCO‐NH_4_I_3_ film with precursor concentration of 0.75 m and preheating temperature of 140 ℃ has been selected for the MN‐PENG design. The output performance of the MN‐PENG measured by an external mechanical system can provide periodic and controllable strain. Output characteristics of the MN‐PENG with unpoled and poled condition (75 kV cm^–1^) under the strain of 0.55% are shown in Figure 3b,c, respectively. It can be observed that both output current and voltage show a significant enhancement after the poling process, which can be ascribed alignment of dipoles within the MDABCO‐NH_4_I_3_ film under electric field.^[^ ^52^ ^]^ The open‐circuit voltage (*V*_OC_) and short‐circuit current (*I*_SC_) of the unpoled MN‐PENG are *V*_OC_ = 9.6 V and *I*_SC_ = 38.3 nA, while those of the poled MN‐PENG are *V*_OC_ = 15.9 V and *I*_SC_ = 54.5 nA, respectively. The comparison of performances among various precursor concentrations and preheating temperatures has also been shown in Figures S6 and S7 (Supporting Information), respectively. In Figure S6 (Supporting Information), the results show that the output voltage and current of MN‐PENG increase with precursor concentration from 0.25 to 0.75 m. However, the output voltage and current dramatically decrease from 0.75 to 1.0 m. This can possibly be attributed to the high leakage current induced by pinholes in the 1.0 m sample (Figure 1d). The output performances of MN‐PENG also increase with the preheating temperature, as shown in Figure S7 (Supporting Information). This shows that high crystal quality and reduced grain boundaries in samples can be successfully achieved with high preheating temperature, leading to better device performance.

To evaluate the relation between the applied strain and the output performance of MN‐PENG, the dependence of applied strain on output performance is shown in Figure 3d. The voltage and current increase from 7.1 to 15.9 V and from 34.8 to 54.5 nA, respectively, as the strain increases from 0.29% to 0.55%. The results suggest that the MN‐PENG is of great potential to be a self‐powered strain sensor. The details in defining *ε* (%) for strain measurement are shown in Figure S8 and Table S3 (Supporting Information). Figure 3e displays the output current and power density of the MN‐PENG with external resistance load under the strain of 0.55%. The corresponding schematic circuit diagram is shown in Figure S9 (Supporting Information). The peak power density of MN‐PENG can reach 2 mW m^−2^ under an external load of 250 MΩ. The output voltages with different external load resistances under the strain of 0.55% have also been investigated and shown in Figure S10 (Supporting Information). Figure 3f demonstrates the excellent stability of poled MN‐PENG under the strain of 0.55% with no significant degradation observed for over 5000 bending cycles, the entire distribution of output signals obtained from the test was also shown in Figure S11 (Supporting Information).

To further confirm the output generated purely from the MN‐PENG, a switching polarity measurement has been performed and shown in Figure S12 (Supporting Information). The output voltage and current exhibit the same magnitude and phase, indicating that the output signals are originated from the MN‐PENG.^[^ ^53^, ^54^ ^]^ In addition, the output signals from a control sample (without MDABCO‐NH_4_I_3_) and a unpoled MN‐PENG (with MDABCO‐NH_4_I_3_) are compared and shown in Figure S13 (Supporting Information), suggesting the origin of piezoelectricity from the MDABCO‐NH_4_I_3_ film. The distribution of piezoelectric potential in the MN‐PENG has been simulated by finite element analysis (FEA), showing the piezoelectric signals mainly come from the MDABCO‐NH_4_I_3_ film (Figure S14, Supporting Information). Figure 3g,h shows the dependence of output voltage and current on the applied frequencies. The output voltage and current can reach ≈16 V and ≈0.6 µA under an applied frequency of 3 Hz and a strain condition of 0.55%. The output voltage remains constant despite different applied frequencies, while the output current increases with the applied frequency. This shows that the MN‐PENG can be utilized to harvest ambient mechanical energy with various frequencies. As shown in Figure 3i and Video S1 (Supporting Information), the MN‐PENG has been applied to light up a commercial green light‐emitting diode (LED) without using any capacitor, demonstrating the potential of MN‐PENG for practical applications. In addition, the MN‐PENG has also been applied to charge a capacitor. As shown in Figure S15 (Supporting Information), the MN‐PENG can charge a 4.7 µF capacitor up to 4.8 V within 7 min, showing outstanding biomechanical energy‐harvesting capability.

**Figure 4:**
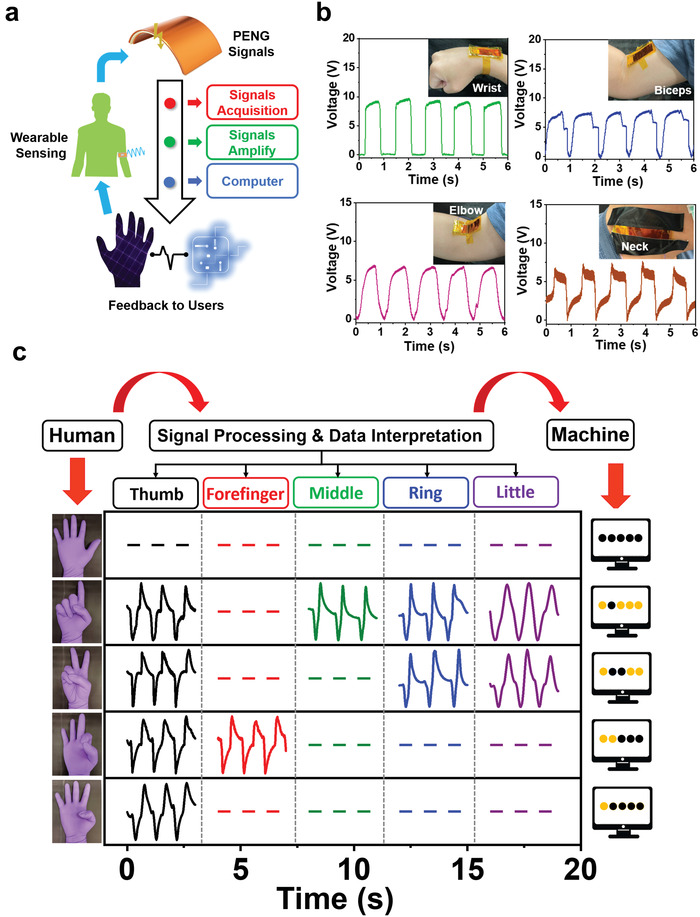
Demonstrating of a human–machine interface application by MN‐PENGs. a) Schematic illustration of the human–machine interface operation. b) Voltage signals from wrist, biceps, elbow, and neck. c) Signals in the smart gesture recognition system.

Self‐powered sensing systems based on PENGs with interactive and communicative functions have become popular for human–machine interface applications.^[^ ^55^, ^56^ ^]^ Herein, a schematic illustration of the human–machine interface based on the MN‐PENG is demonstrated in **Figure** **4**a. By acquiring and amplifying signals from the MN‐PENG, the feedback signal can be provided for immediate interactions between human and machine. Figure 4b shows the output voltage detected from various body motions including wrist, elbow, biceps, and neck, indicating the capability of the MN‐PENG for harvesting mechanical energy from various body parts. Meanwhile, to further investigate the feasibility of using MN‐PENG in harvesting energy and detecting signals from localized physiological motions, the MN‐PENG was utilized to record signals generated from human pulse shown in Figure S16 (Supporting Information). Figure S17 presents the bending‐angle dependent output voltage from 30° to 120°, where the corresponding output voltage range from 1.14 to 10.38 V. Meanwhile, a smart gesture recognition system has been designed by combining five MN‐PENGs (Figure 4c). The relative output signals of five MN‐PENGs have been evaluated before combing with the glove for system integration. It is shown in Figure S18 that similar output voltage and current can be obtained. Five MN‐PENGs were marked number 1, 2, 3, 4, and 5 indicating thumb, forefinger, middle, ring, and little fingers on the glove. Five gestures including “one,” “two,” “three” “four,” and “five” can be successfully displayed in real‐time operation by obtaining the output voltage signals from the MN‐PENGs. The output signals were further transferred into a series of visualized symbols on computer interface denoted as yellow and black circles. The yellow circles represent the bending state, while the black circles represent the unbending state. Figure S19 shows the output signals of gesture “five” defined as the original state, where all fingers with MN‐PENGs were set as the unbending state. The real‐time demonstration is also shown in Video S2 (Supporting Information). The results indicate that the MN‐PENGs are of great potential for future self‐powered sensor and human–machine interaction platform designs.

**Figure 5:**
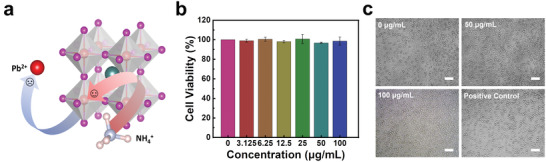
Cytotoxicity of the as‐synthesized MDABCO‐NH_4_I_3_ (*n* = 9). a) Schematic of replacing Pb^2+^ with NH_4_^+^ as the B‐site cation. b) Cell viability of L929 fibroblasts with various concentrations of MDABCO‐NH_4_I_3_ in the culture medium measured by the CCK‐8 assay. c) Phase contrast images of L929 cells cultured in 96‐well plates with various concentrations of MDABCO‐NH_4_I_3_. The scale bar is 100 µm.

As for perovskite‐based PENGs, the existence of lead contents remains a critical factor to be concerned that limits their applications, especially for skin electronics and in vivo sensing technology. Previously, some attempts have been reported to address the cytotoxicity of lead‐containing perovskite materials.^[^ ^57^ ^]^ Benmessaoud et al. performed a cytotoxicity evaluation of methylammonium lead iodide (MAPbI_3_) on primary neurons and neuroblastoma cells. The results demonstrated a massive apoptotic cell death occurred once primary neurons and neuroblastoma cells were exposed to MAPbI_3_.^[^ ^57^ ^]^ Quaroni et al. reported the cytotoxicity of MAPbI_3_ and methylammonium tin iodide (MASnI_3_) evaluated by SH‐SY5Y cell and A549 cell tests, respectively. The results indicated that the higher concentration of perovskite material inside the cell medium, the lower cell viability rate observed.^[^ ^58^ ^]^ As mentioned above, the role of B‐site Pb^2+^ cation was replaced by the NH^4+^ cation in MDABCO‐NH_4_I_3_ to reduce toxicity, as illustrated in **Figure** **5**a. To further assess the cytotoxicity of the as‐synthesized MDABCO‐NH_4_I_3_ film, a cell viability test has been conducted by using L929 fibroblasts.^[^ ^23^, ^59^ ^]^ In Figure 5b, the cell viability of control (0 µg mL^−1^) was set as 100%, while the medium containing 10% DMSO was used as the positive control. The cell viability of the group with maximum MDABCO‐NH_4_I_3_ concentration at 100 µg mL^−1^ is 98.49 ± 4.30%, which is similar to the control group. In addition, the distribution of the cell viability is uniform among different concentrations of MDABCO‐NH_4_I_3_. All the results have been averaged over 9 independent runs (n = 9). The cell morphologies of L929 fibroblasts with different concentrations of the MDABCO‐NH_4_I_3_ film, which include 0, 50, 100 µg mL^−1^, and 10% DMSO (positive control) are shown in Figure 5c. No significant cytotoxicity has been observed in the presence of MDABCO‐NH_4_I_3_. These results indicate the negligible cell toxicity of the as‐synthesized MDABCO‐NH_4_I_3_ for L929 fibroblasts. All results of cell morphology and cell viability are also shown in Figures S20 and S21 (Supporting Information).

**Figure 6:**
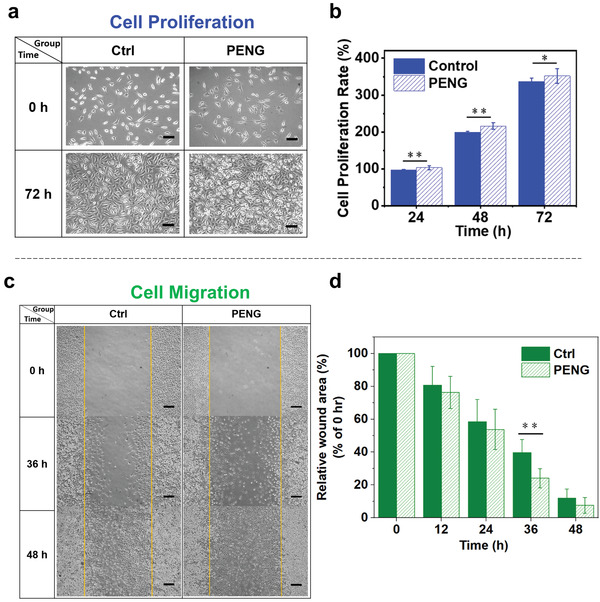
In vitro cellular behaviors of L929 fibroblasts under electrical stimulation by the MN‐PENG. a) Cell morphologies at 0 and 72 h without (control) and with ES from MN‐PENG (PENG). The scale bar is 100 µm. b) Proliferation rates of L929 cells stimulated by the MN‐PENG and control cells at 24, 48, and 72 h (*n* = 3, *: *p* < 0.05, and **: *p* < 0.01). c) Scratched areas of the L929 fibroblasts in control and PENG stimulation groups at 0, 36, and 48 h. The scale bar is 200 µm. d) Quantitative analysis of the migration results (*n* = 5, *: *p* < 0.05, and **: *p* < 0.01).

Electrical stimulation therapy has been regarded as a safe and convenient method for the treatment of several diseases in recent years, especially in the field of regenerative medicine and neurology such as wound healing, neuroplasticity, and neuro repairing.^[^ ^23^, ^59^, ^60^, ^61^ ^]^ For wound healing, the concept came from the endogenous electric fields, which can guide fibroblasts to migrate to the wound area.^[^ ^62^, ^63^, ^64^, ^65^ ^]^ Specifically, it has been reported that cell proliferation and migration can be efficiently enhanced by electric fields, providing the benefits for wound healing.^[^ ^22^, ^23^, ^60^, ^62^, ^63^, ^64^, ^65^ ^]^ To exploit the potential of MN‐PENG for cell proliferation and migration, the cellular behaviors of L929 fibroblasts under an electrical stimulation by MN‐PENG have been studied. In Figures S22 and S23 (Supporting Information), the rectified output performance of MN‐PENG and applied force generated by the motor in triggering electrical stimulation tests were also illustrated. Herein, the cells without stimulation were set as control groups. **Figure** **6**a shows the cell morphologies of control and stimulated cells at 0 and 72 h. The results indicate that the cell proliferation behavior was significantly enhanced under ES by MN‐PENG. In Figure 6b, the proliferation rates between the control and stimulated cells at 24, 48, and 72 h show statistical significance. At 72 h, the proliferation rate of stimulated cells is 351.82 ± 19.90%, which is significantly higher than that of control cells (337.06 ± 9.55%). Figure 6c shows the images of in vitro migration assays of both control and experimental cells. As compared to the control group, the migration of L929 fibroblasts has been enhanced toward the center region of wound area by ES from MN‐PENG. In Figure 6d, the statistical results of the wound area in percentage show significantly decrease at 36 h in the MN‐PENG group as compared to the control group. At 36 h, the relative wound areas of stimulated group and the control group are 24.07 ± 5.84% and 39.56 ± 8.07%, respectively. At 48 h, the stimulated group shows the excellent wound recovery with merely 7.56 ± 4.77% wound area, as compared to the wound area of 11.99 ± 5.63% for the control group. The results suggest that the ES from MN‐PENG can significantly promote cell proliferation and enhance cell migration.

## 3 Conclusion

In summary, we have fabricated a MN‐PENG using metal‐free perovskite (MDABCO‐NH_4_I_3_) film for the first time. The piezoelectric and ferroelectric properties of the MDABCO‐NH_4_I_3_ film have been studied to exhibit a piezoelectric constant of 12.81 pm V^−1^ and a remnant polarization of 13.3 µC cm^−2^. The output voltage and current of the MN‐PENG can reach 15.9 V and 54.5 nA, respectively, after the poling process. Moreover, the MN‐PENG is capable of lighting up a commercial LED, charging a capacitor, and serve as a self‐powered strain sensor for a smart human–machine interface platform, demonstrating its feasibility for practical electronics. Furthermore, the MN‐PENG has also been designed as an in vitro electrical stimulation device, which is promising for future portable wound healing system design. These findings not only reveal the great potential of the MDABCO‐NH_4_I_3_ film in toxic‐free, wearable, interactive, and multifunctional smart devices, but also extend the future aspects of utilizing metal free perovskites for biomedical applications.

## 4 Experimental Section

### Synthesis of MDABCO‐NH_4_I_3_ Crystals

1.84 g (15.57 mmol) of 1,4‐diazabicyclo [2.2.2] octane (DABCO, 95%, KANTO, Tokyo, Japan) was first dissolved in 20 mL of acetone (99%, Union Chemical Works Ltd., Hsinchu City, Taiwan) and stirred in an ice bath, then slowly added in 1 mL (15.57 mmol) of iodomethane (CH_3_I, 95%, Showa Chemical Co. Ltd., Tokyo, Japan) for methylation reaction. The white precipitate of methylation DABCO (MDABCO‐I) was collected and dried. Subsequently, 3.96 g (15.57 mmol) of MDABCO‐I was dissolved into 20 mL DI water and stirred in an ice bath. 3.5 mL (26.51 mmol, overdose) of hydriodic acid (HI, 57%, Alfa Aesar, MA, USA) was then slowly added for the protonation reaction. The precipitate of MDABCO‐I_2_ was formed and rinsed by methanol and acetone to remove excess hydriodic acid until the color became white or slightly yellow. The MDABCO‐I_2_ was then dried up and recrystallized for 1 time without further purification. 0.365 g (1 mmol) of MDABCO‐I_2_ and 0.145 g (1 mmol) NH_4_I (99%, Union Chemical Works Ltd., Hsinchu City, Taiwan) were dissolved in 20 mL DI water and slowly evaporated at 55 ℃ to form the MDABCO‐NH_4_I_3_ crystals.

### Preparation of the PEDOT:PSS Film

PEDOT:PSS (Clevios PH1000) was first filtered and mixed with 2 vol% of DMSO (99%, Acros Organics, NJ, USA) and 0.05 wt% of Triton X‐100 (99%, Alfa Aesar, MA, USA). 200 µL of the PEDOT:PSS solution was then spin‐coated on the polyimide (PI) substrate at 1000 rpm for 15 s. The sample was then annealed under 120 ℃ for 10 min. The area and the thickness of the PEDOT:PSS film were 1.5 × 1.5 cm^2^ and 75 nm, respectively.

### Preparation of the MDABCO‐NH_4_I_3_ Film

Various concentrations (0.25, 0.5, 0.75, and 1.0 m) of MDABCO‐NH_4_I_3_ solutions were prepared by dissolving 0.133 g (0.25 mmol), 0.266 g (0.5 mmol), 0.398 g (0.75 mmol), and 0.531g (1 mmol) of MDABCO‐NH_4_I_3_ crystals into 1 mL deionized water, respectively. The MDABCO‐NH_4_I_3_ film was prepared by using a hot‐casting method. The PI substrate with PEDOT:PSS film was first preheated at a certain temperature for 10 min and quickly moved onto the spin‐coater. 150 µL of the perovskite solution was dropped onto substrate and spin‐coated under 3000 rpm for 15 s. After spin‐coating, the intermediate film was annealed under 80 ℃ for 10 min.

### Material Characterizations

The XRD patterns were characterized by using a Bruker D2 phaser diffractometer at 30 kV and 10 mA (Cu K*α* with 1.54184 Å). The SEM images were characterized by a JEOL JSM‐7900F SEM operated at an acceleration voltage of 5 kV. The leakage current and ferroelectric hysteresis loop of the films were measured by Keithley 2612B sourcemeter on the samples coated with 50 nm of nickel as the top electrode (0.2 × 0.3 cm^2^). PFM measurements were characterized by using a Bruker Dimension Icon Atomic Force Microscope under contact mode with a tunable LS PR AC bias and a driving frequency of 15 kHz. The amplitude and phase response loops were scanned with ‐10 to +10 V DC bias.

### Fabrication of the MN‐PENG Device

Two pieces of PI substrates each with an area of 2 × 6 cm^2^ were deposited with 100 nm of silver (Ag) as top and bottom electrodes by a radio‐frequency sputtering system (Kao Duen Technology Co., New Taipei City, Taiwan). The top electrode was fully covered with Ag, and bottom electrode was coated with 2 × 2 cm^2^ of Ag to contact with the PEDOT:PSS film and prevent the reaction between Ag and halide ions. The PEDOT:PSS and the MDABCO‐NH_4_I_3_ films were deposited near the center of the substrate with an area of 1.5 × 1.5 cm^2^. PDMS resin (Sil‐More Industrial Ltd., New Taipei City, Taiwan) was mixed with the curing agent (weight ratio of 10:1) and placed in a 60 ℃ oven for 20 min to partially remove the ethylbenzene in the curing agent. The mixture was then spin‐coated onto the other Ag‐coated PI substrate (top electrode) and partially dried at 60 ℃ for 20 min. Finally, the pieces were bonded and kept in a 60 ℃ oven for 2 h. The final MN‐PENG device was formed by connecting electrodes and copper wires by silver gel and sealing the whole device with Kapton tape to eliminate gaps. For the electrical poling procedure, an external electric field (75 kV cm^−1^) was applied to the device at room temperature for 2 h.

### Characterization of the MN‐PENG Device

The output characteristics of the MN‐PENG were measured with Keithley 6514 electrometer (200 TΩ input impedance). A commercial linear mechanical system was used for providing controllable and periodically bending strain. As for the experiemnt of using MN‐PENG as a wearable sensor to dectect human motion, no approvals were required for the experiement, which complied with all relevant regulations.

### Cell Culture and Viability Test

The L929 fibroblast cell line was purchased from Bioresource Collection and Research Center (BCRC, Hsinchu City, Taiwan). Cells were maintained in Dulbecco‘s Modified Eagle Medium (DMEM, Corning, NY, USA) supplemented with 10% fetal bovine serum (FBS, Corning, NY, USA) and 1% antibiotic of penicillin‐streptomycin solution (Corning, NY, USA) at 37°C in a 5% CO_2_ incubator (BB15, Thermo Fisher Scientific, MA, USA). To investigate the cytotoxicity of MDABCO‐NH_4_I_3_, 1 × 10^4^ cells of L929 fibroblasts were seeded in a 96‐well cell culture plate (Cat. No. 310109008, Thermo Fisher Scientific, MA, USA) and incubated at 37 ℃ in the 5% CO_2_ incubator overnight. Then, the media were replaced with various concentrations of MDABCO‐NH_4_I_3_ and further incubated for 24 h. Cell viability was determined by the Cell Counting Kit‐8 (CCK‐8, Dojindo Laboratories, Kumamoto, Japan). The absorbance at the wavelength of 450 nm was measured by a microplate spectrophotometer (Multiskan GO, Thermo Fisher Scientific, MA, USA). Images of the cell morphologies were obtained by using an inverted optical microscope (Olympus CK30, Olympus, Tokyo, Japan).

### Cell Proliferation and Migration

To investigate the cell proliferation induced by the electric stimulation of MN‐PENG, 1 × 10^5^ cells of L929 fibroblasts were seeded in 35 mm diameter culture dishes (Figure S24, Supporting Information) for 24 h. Cells were regularly stimulated for 1 h d^−1^. Cell morphologies were obtained by using an inverted optical microscope (Olympus CK30). The cell proliferation rate was evaluated by Cell Counting Kit‐8 at 24, 48, and 72 h. Cell migration was characterized by evaluating an in vitro scratch assay. Cells were seeded in 35 mm diameter culture dishes (1 × 10^6^ cells per dish) and grown at 37 ℃ in 5% CO_2_ incubator overnight. Confluent cells were maintained in DMEM containing 5% FBS. A straight scratch was made by using a steriled 1000 µL tip before MN‐PENG stimulation. Cells were regularly stimulated for 1 h d^−1^. The scratched regions were recorded by an inverted optical microscope (Olympus CK30) and the areas were calculated by using the ImageJ software (NIH, Bethesda, MD, USA).

### Statistical Analysis

Each experiment was repeated three times. The cell proliferation rate and the relative wound area were expressed as mean ± standard deviation (SD). The statistical analysis was performed by using the SPSS software (version 27.0.1.0, IBM SPSS, IL, USA). All results were analyzed by the two‐tailed t‐test, where *p* < 0.05 was considered statistically significant.

## Conflict of Interest

The authors declare no conflict of interest.

## Supporting information

Supporting information

Supporting information

Supporting information

## Data Availability

Research data are not shared.
