# Supplementary material for: Metal‐Free Perovskite Piezoelectric Nanogenerators for Human–Machine Interfaces and Self‐Powered Electrical Stimulation Applications
Source: Adv Sci (Weinh). 2022 Apr 20;9(18):2105974. doi: 10.1002/advs.202105974 (PMC9218782; doi:10.1002/advs.202105974)
Supplement: Supplementary file 1 — Supporting information [file ADVS-9-2105974-s002.pdf]

ADVANCED SCIENCE
Open Access

Supporting Information

for *Adv. Sci.*, DOI 10.1002/advs.202105974

Metal-Free Perovskite Piezoelectric Nanogenerators for Human–Machine Interfaces and Self-Powered Electrical Stimulation Applications

Han-Song Wu, Shih-Min Wei, Shuo-Wen Chen, Han-Chi Pan, Wei-Pang Pan, Shih-Min Huang, Meng-Lin Tsai and Po-Kang Yang**

Supporting Information

Metal-Free Perovskite Piezoelectric Nanogenerators for Human-Machine Interfaces and Self-Powered Electrical Stimulation Applications

Han-Song Wu, Shih-Min Wei, Shuo-Wen Chen, Han-Chi Pan, Wei-Pang Pan, Shih-Min Huang, Meng-Lin Tsai, and Po-Kang Yang**

Table S1. FWHMs of the XRD (200) peaks for MDABCO-NH_4I_3 films prepared by various preheating temperatures.

Preheating Temperature (°C)	FWHM (degree)
RT	0.160
100	0.127
120	0.127
140	0.114

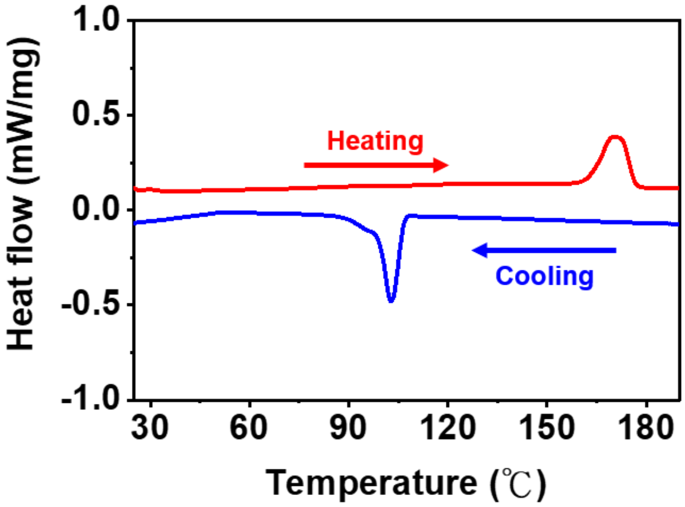

Figure S1. DSC characteristic of MDABCO-NH_4I_3 thin film.

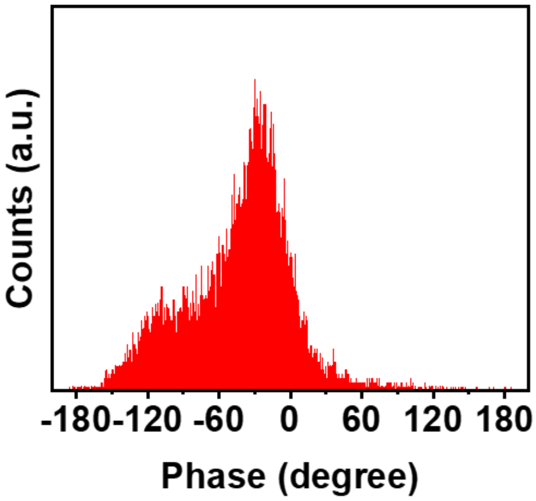

Figure S2. Phase distribution in phase image

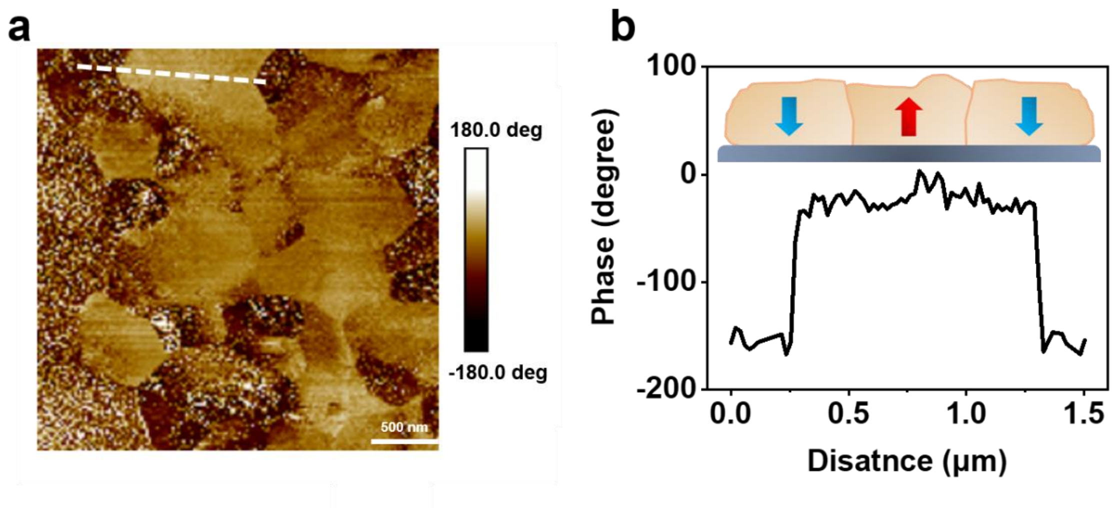

Figure S3. a) The chosen position in phase image (dash line) and b) Corresponding phase profile.

Table S2. The piezoelectric coefficient (d_{33}) measured from various points and the average value.

Point	d_{33} (pm/V)
1	12.848
2	12.744
3	12.741
4	12.769
5	12.928
Average	12.806

The calculation method of the piezoelectric coefficient (d_{33}) is based on the following equation.[S1]

$$d_{33} = \frac{Amplitude\ (mV) \times deflection\ sensitivity\ (nm/V)}{Gain \times AC\ bias\ (mV)} \quad (1)$$

The d_{33} coefficient was performed by using the static-sensitivity-based quantification method based on evaluating the reciprocal slope of the force-distance curve and making it as quantification factor of the amplitude from the PFM measurement.[S2,S3] The observed sensitivity was 118.72 nmV^{-1}. The sensitivity depends on the cantilever system and the tip selected.

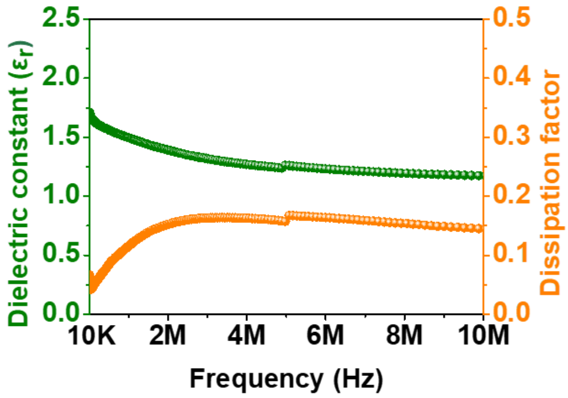

Figure S4. Frequency dependent relative dielectric constant and dissipation factor. The dielectric constant plays an important role in piezoelectric coefficient through the following formula: $d_{33} = 2Q_{33} \times \varepsilon_{33} \times P_r$. ($d_{33}$: piezoelectric coefficient, Q_{33}: electrostriction constant, ε_{33}: relative dielectric constant, P_r: remanent polarization).[S4]

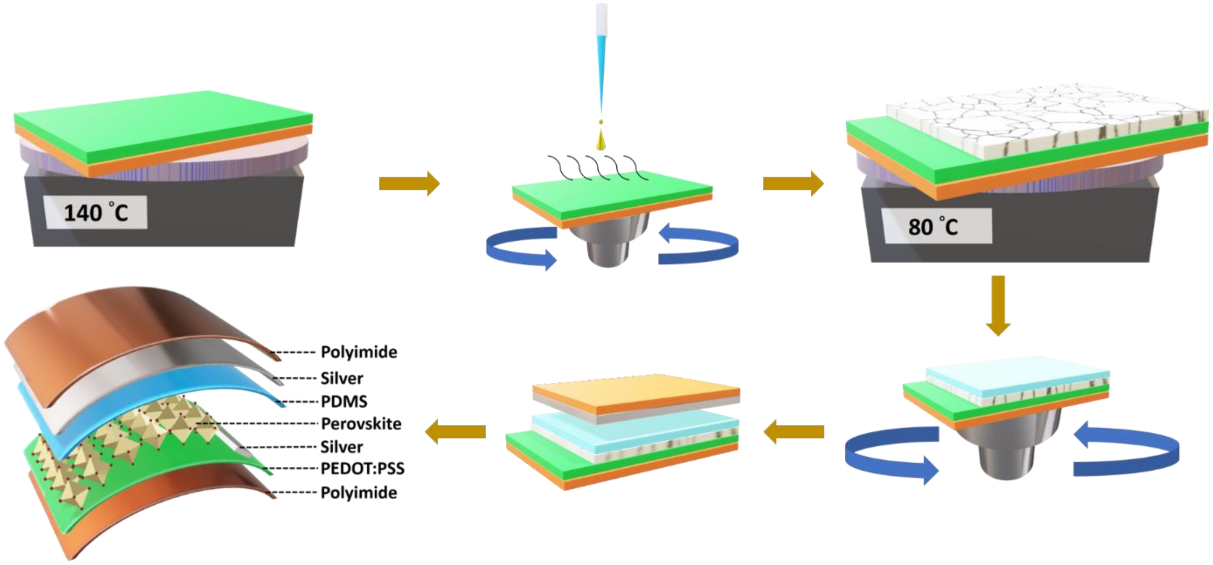

Figure S5. Schematic illustration of the MN-PENG fabrication procedures.

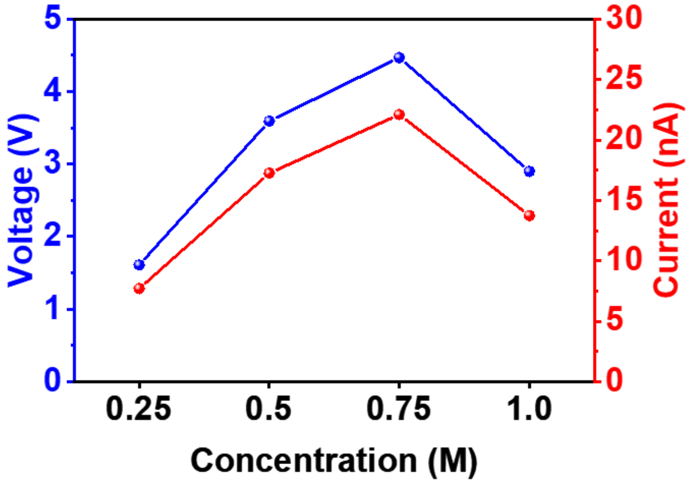

Figure S6. Output characteristics of the MN-PENG devices fabricated with various precursor concentrations (without preheating).

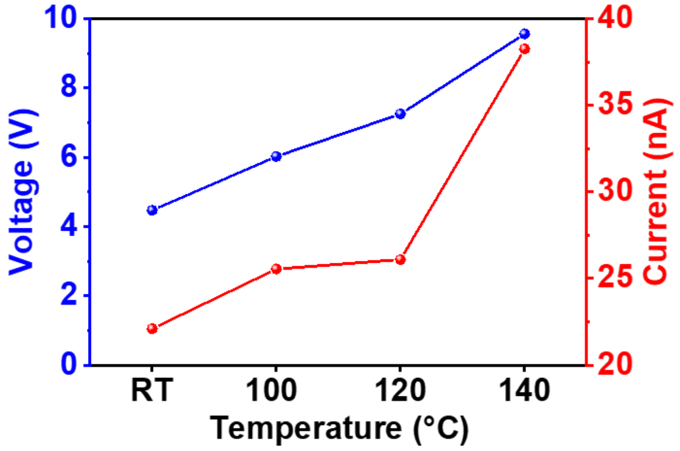

Figure S7. Output characteristics of the MN-PENG devices fabricated with various preheating temperatures (under fixed precursor concentration of 0.75 M).

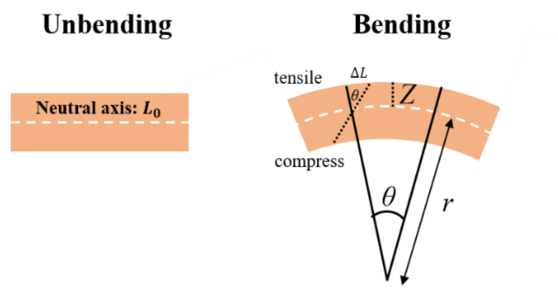

Tensile: $\boldsymbol{\varepsilon > 0}$

Compress: $\boldsymbol{\varepsilon < 0}$

$$\boldsymbol{\varepsilon}\ \textbf{(strain)}= \frac{\Delta L}{L} = \frac{L'-L}{L} = \frac{(r+Z)\times\theta - r\times\theta}{r\times\theta} = \frac{Z\theta}{r\theta} = \frac{Z}{r}$$

$$\boldsymbol{\varepsilon}\ (\%) = \frac{\Delta L}{L_0} \times \mathbf{100\%}$$

Figure S8. Illustration of the strain calculation process.

Table S3. Relation between displacement and strain.

Displacement (mm)	Strain (%)
10	0.29
12.5	0.35
15	0.41
17.5	0.50
20	0.55

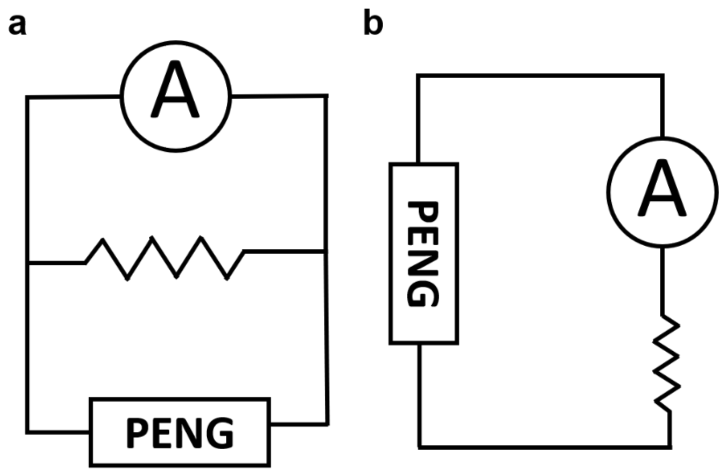

Figure S9. Schematic circuit diagrams for measuring a) output voltage and b) output current under external loads.

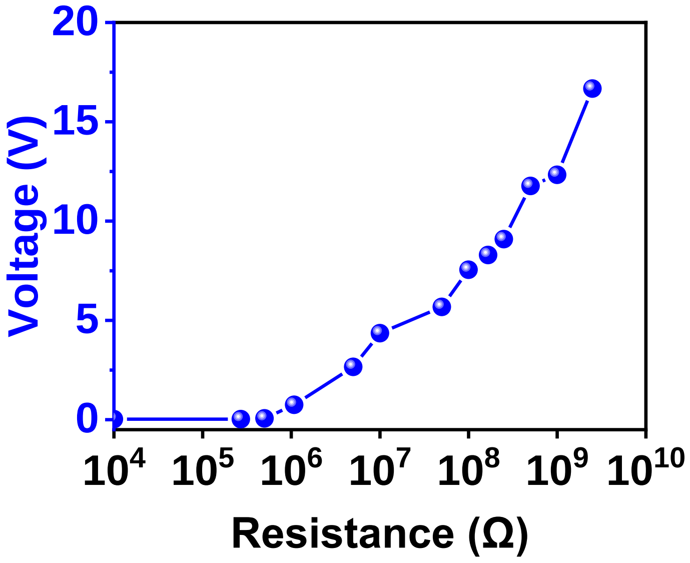

Figure S10. External resistance dependent output voltage of the MN-PENG.

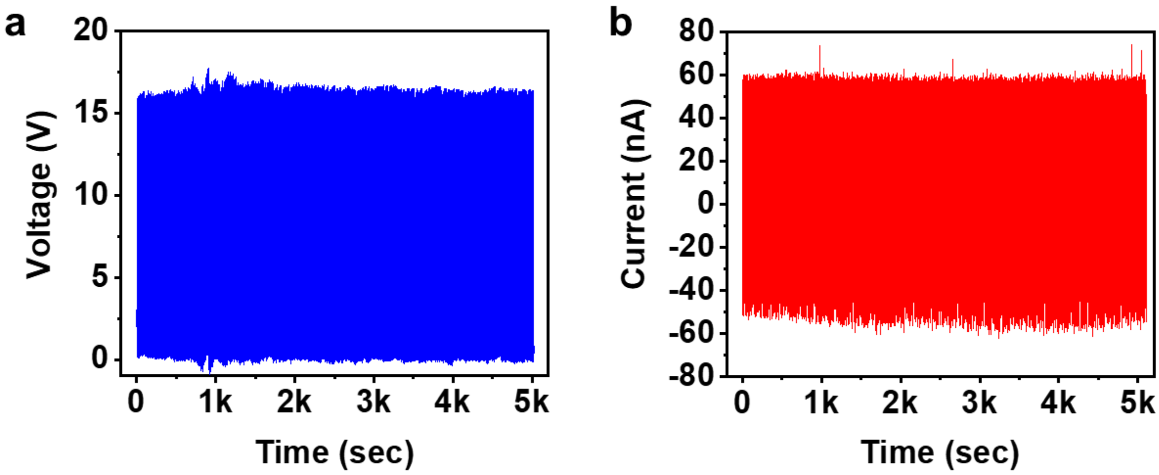

Figure S11. Stability test of the MN-PENG device. a) Output voltage under operation for 5000 s. b) Output current under operation for 5000 s.

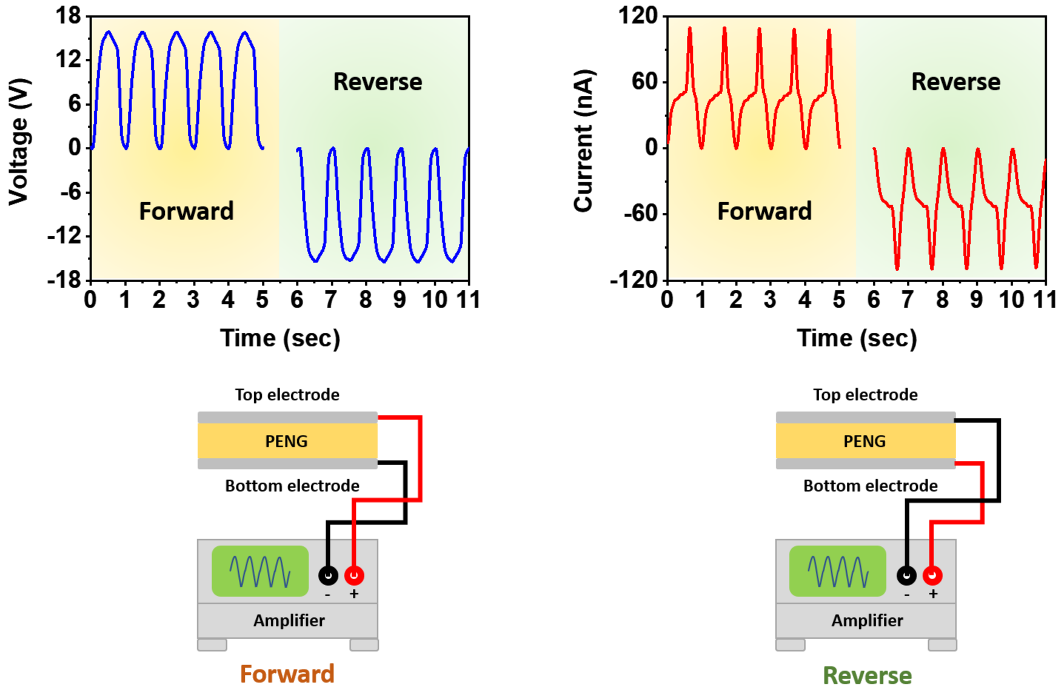

Figure S12. Output characteristics of the MN-PENG with forward and reverse wiring circuits.

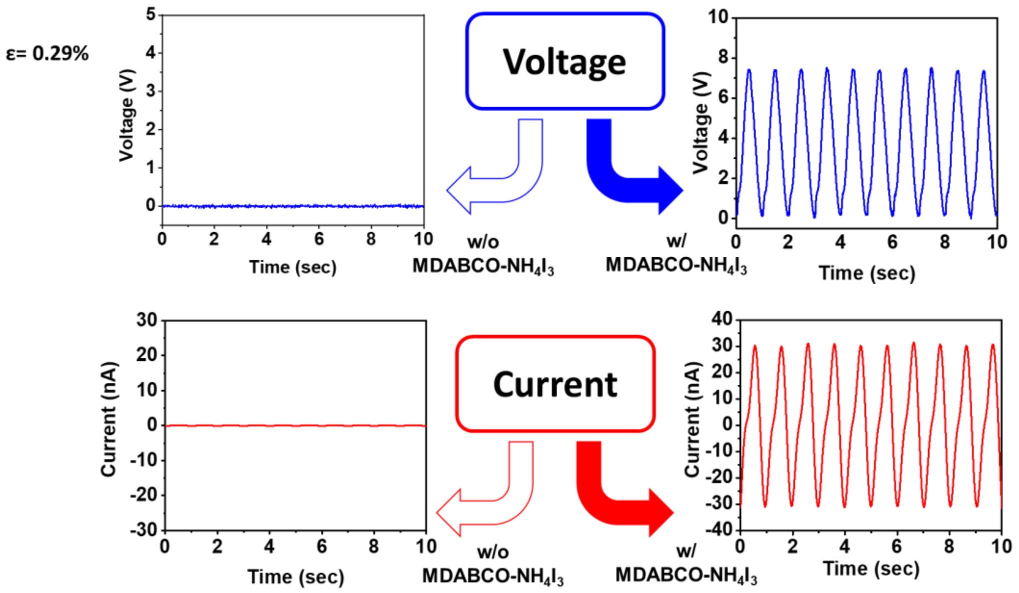

Figure S13. Output performance comparison between the devices with MDABCO-NH_4I_3 and without MDABCO-NH_4I_3.

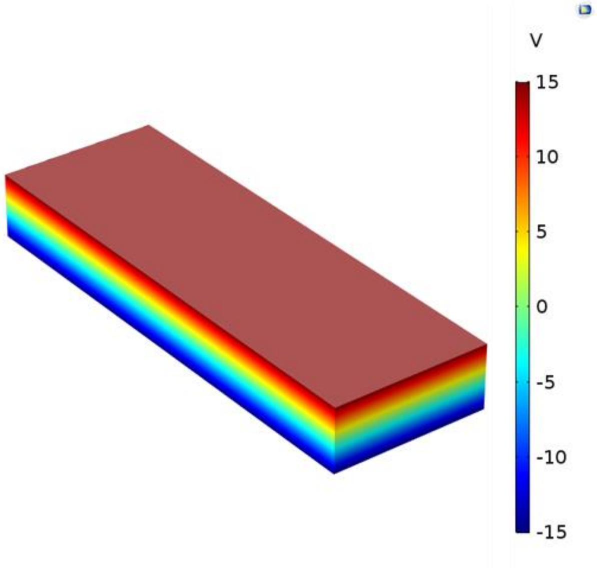

Figure S14. Piezoelectric potential distribution of the MN-PENG.

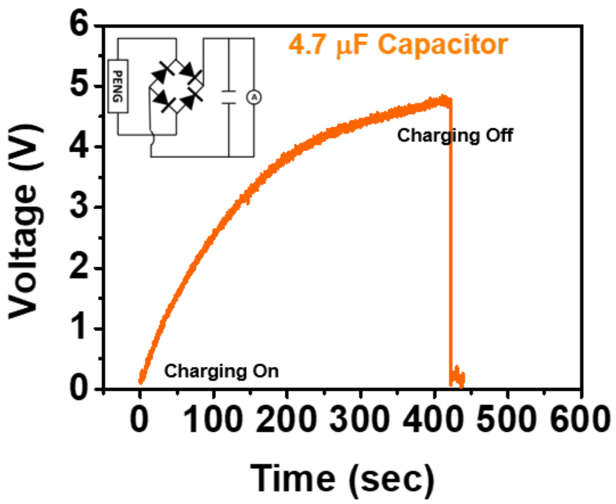

Figure S15. Charging profile of a 4.7 μF capacitor charged by the MN-PENG operated under 1 Hz and ε = 0.55%.

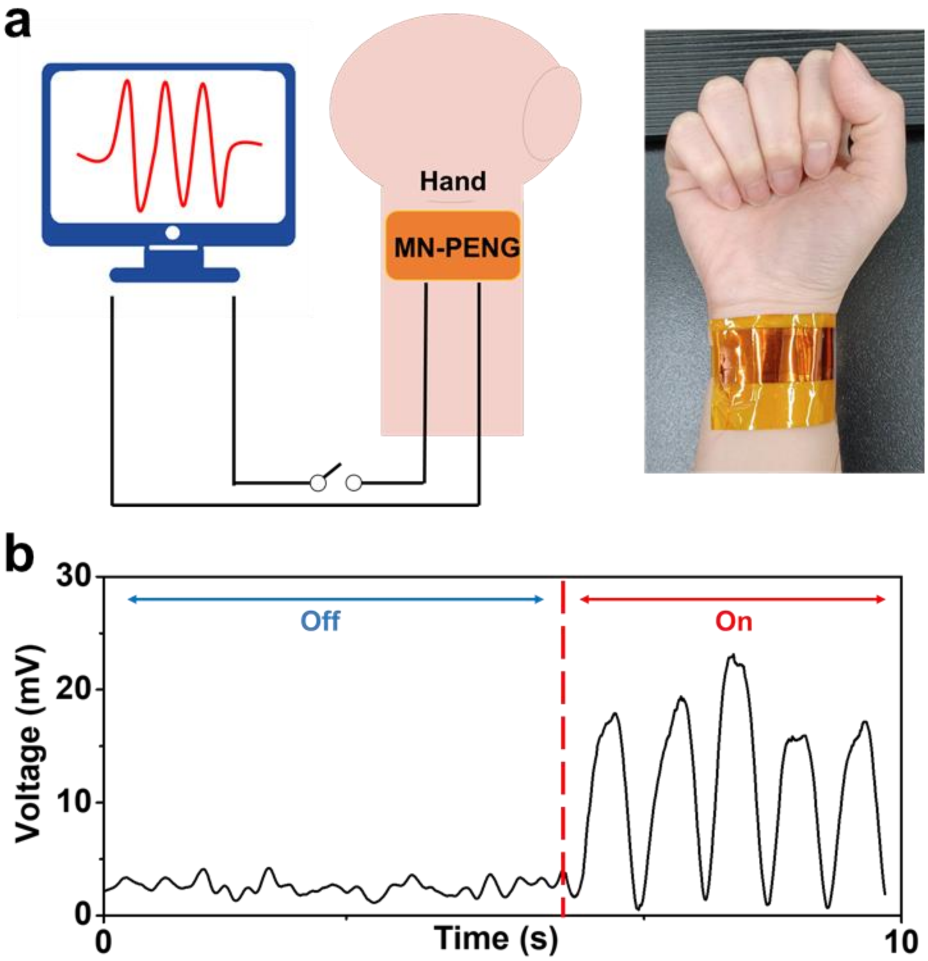

Figure S16. MN-PENG as an *in vitro* pulse sensor device. a) Schematic illustration and photographic image of the *in vitro* pulse sensor device. b) Voltage signals from the human pulse, the exhibited frequency was 1.4 Hz (~84 bpm, bpm: beats per minute), which was similar to the previous literatures. [S5-S7]

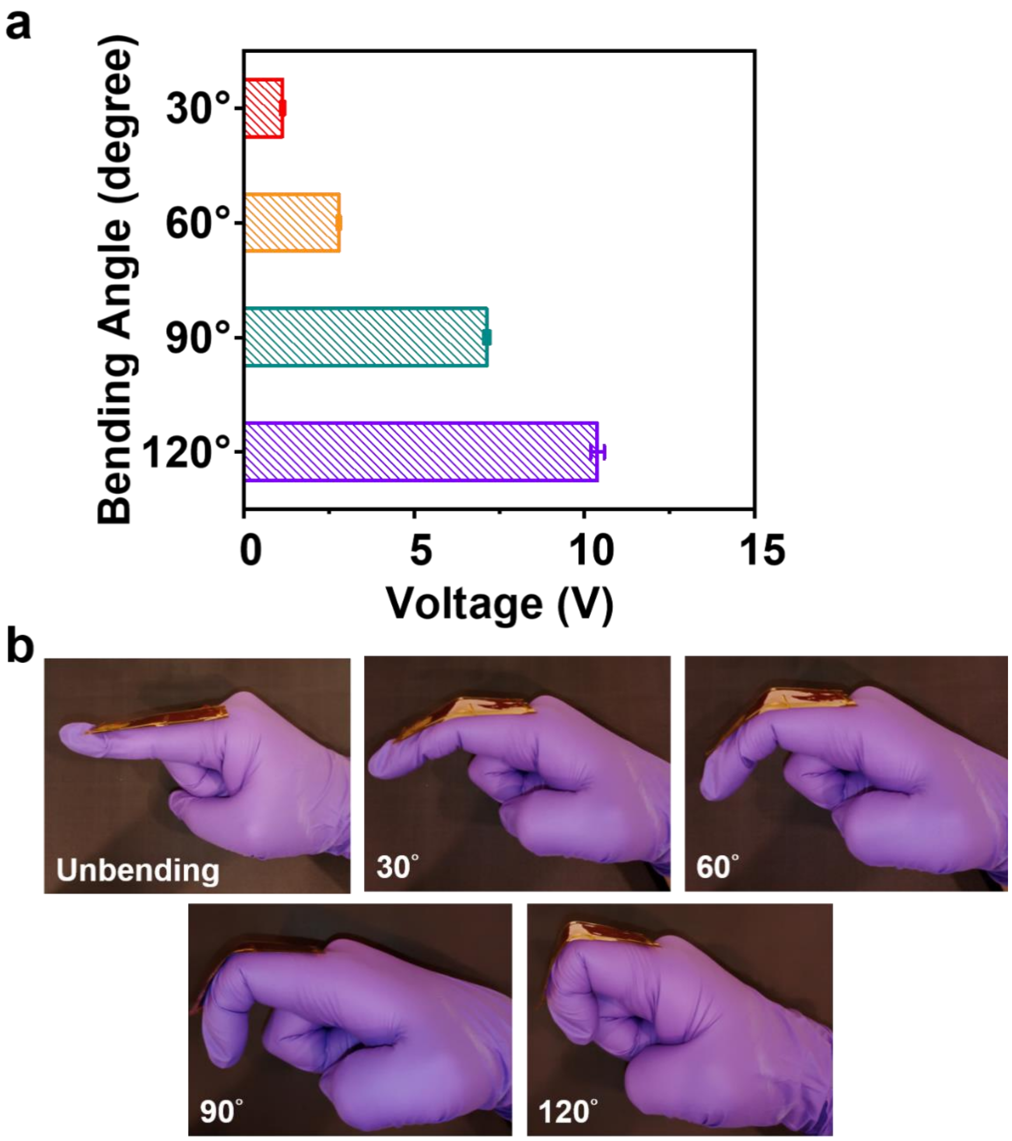

Figure S17. a) Output voltages of the MN-PENG on the forefinger with various bending angles. b) Images of various bending angles operated by the forefinger.

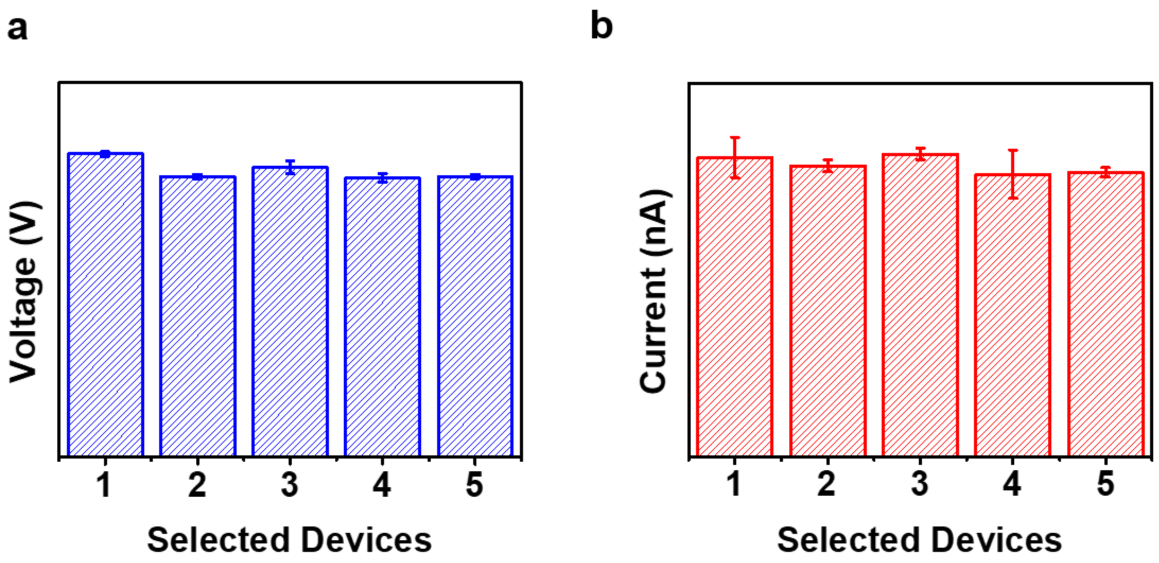

Figure S18. a) Output voltage and b) Output current of 5 MN-PENGs before integrating into the smart gesture system.

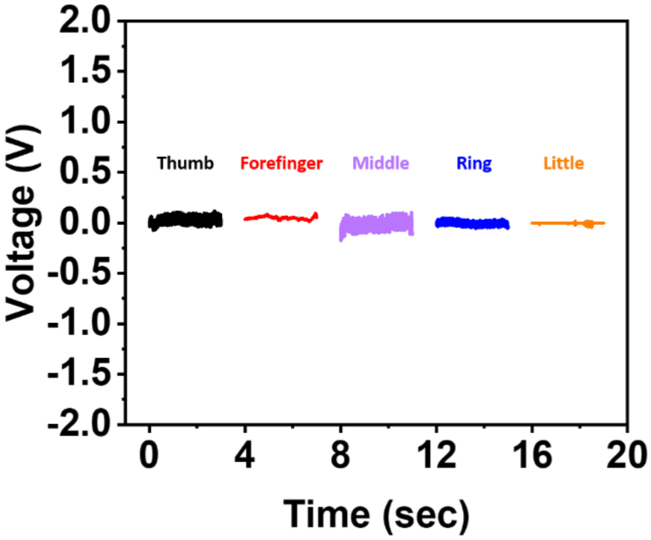

Figure S19. Output voltage of MN-PENG on each finger at the unbending state.

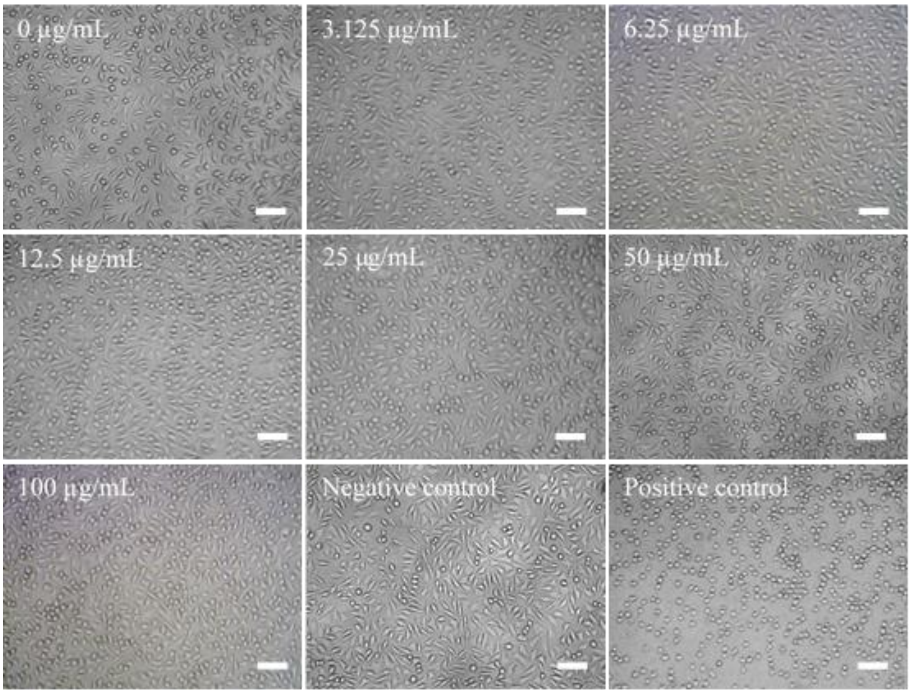

Figure S20. Image of cells cultured in a 96-well plate with various concentrations of MDABCO-NH_4I_3. The media containing 10% DMSO and 100 μL/mL of sterilized water serve as positive control and negative control, respectively. The scale bar is 100 μm.

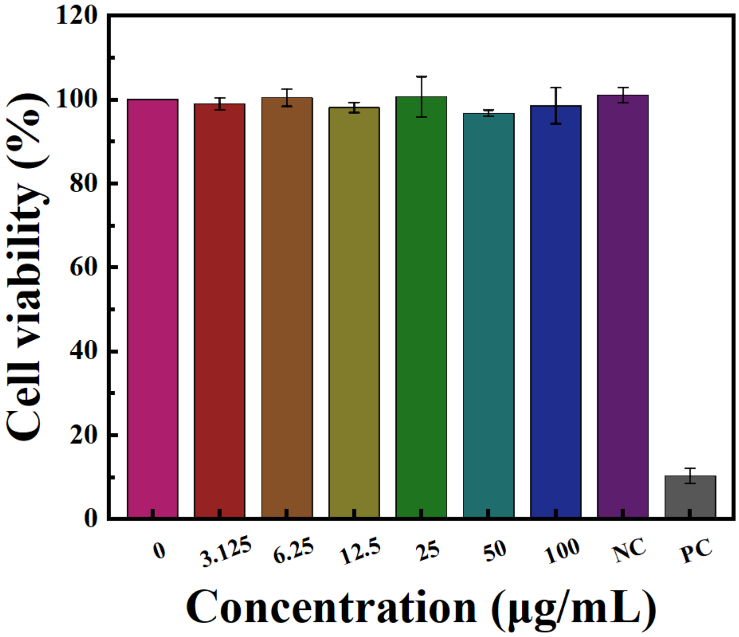

Figure S21. Cell viability with various concentrations of MDABCO-NH_4I_3 (n = 9). The media containing 10% DMSO and 100 µL/mL of sterilized water serve as positive control (PC) and negative control (NC), respectively.

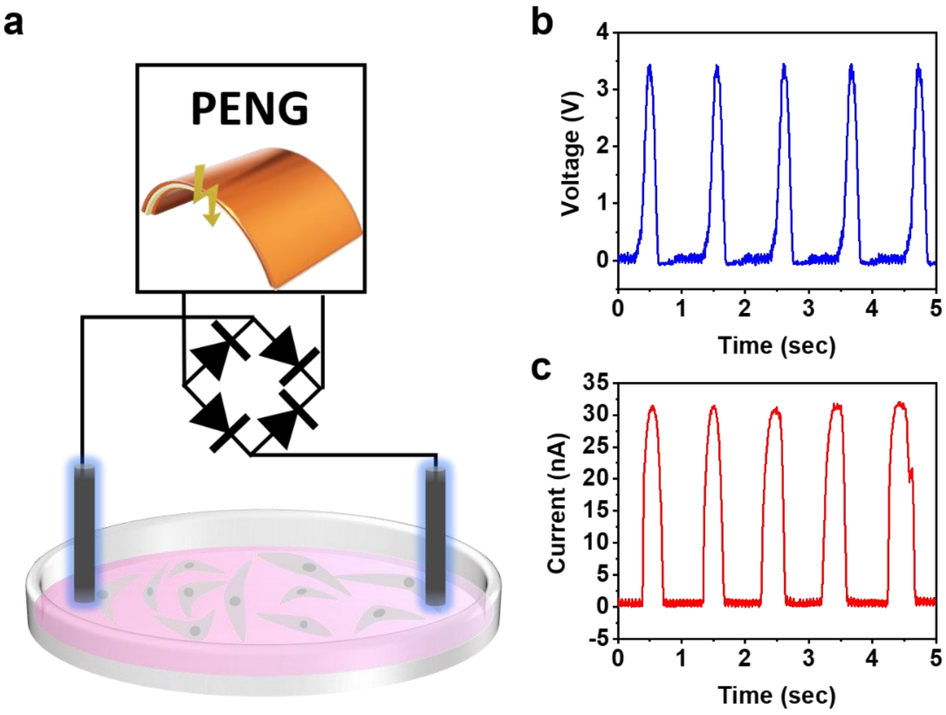

Figure S22. a) Schematic illustration of the *in vitro* electrical stimulation system. The MN-PENG was activated by a homemade mechanical system. b) Output voltage after rectification. c) Output current after rectification.

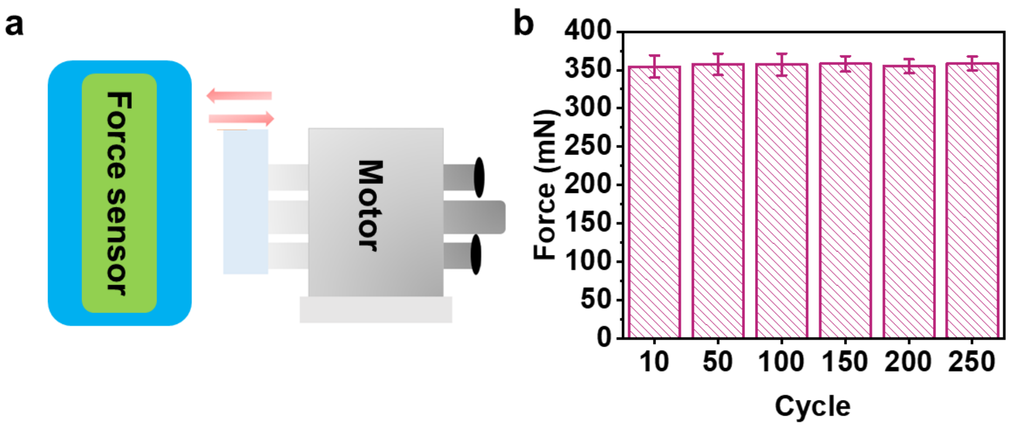

Figure S23. Illustration and distribution of applied force for generating electricity. a) Illustration of force measurement. b) Statistical diagram of force in 250 cycles.

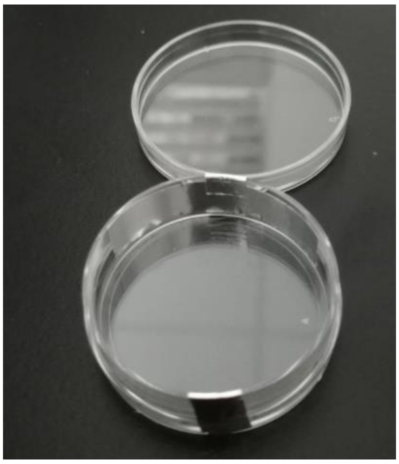

Figure S24. A 35 mm-diameter cell culture dish with symmetric aluminum electrodes capping on the margin.

References

[S1] P. K. Yang, S. A. Chou, C. H. Hsu, R. J. Mathew, K. H. Chiang, J. Y. Yang, Y. T. Chen, *Nano Energy* **2020**, *75*, 104879.

[S2] O. Kwon, D. Seol, H. Qiao, Y. Kim, *Adv. Sci.* **2020**, *7*, 1901391.

[S3] R. Proksch, *J. Appl. Phys.* **2015**, *118*, 072011.

[S4] G. Helke, K. Lubitz, in *Springer Ser. Mater. Sci.*, Vol. 114 (Eds: W. Heywang, K. Lubitz, W. Wersing), Springer, Berlin, Heidelberg, Germany **2008**, Ch. 4.

[S5] R. Avram, G. H. Tison, K. Aschbacher, P. Kuhar, E. Vittinghoff, M. Butzner, R. Runge, N. Wu, M. J. Pletcher, G. M. Marcus, J. Olgin, *npj Digital Med.* **2019**, *2*, 58.

[S6] P. Li, Z. Zhang, W. Shen, C. Hu, W. Shen, D. Zhang, *J. Mater. Chem. A* **2021**, *9*, 4716.

[S7] S. Cheng, S. Han, Z. Cao, C. Xu, X. Fang, X. Wang, *Small* **2020**, *16*, 1907461.
